# Modulating interhemispheric prefrontal dynamics of aggressive behavior: Sex differences and the association with personal disposition

**DOI:** 10.3758/s13415-025-01289-x

**Published:** 2025-04-11

**Authors:** Chiara Gramegna, Maria Franca, Nadia Bolognini

**Affiliations:** 1 PhD Program in Neuroscience, School of Medicine and Surgery, University of Milano-Bicocca, Piazza Dell’Ateneo Nuovo 1, 20126 Milan, Italy; 2 Department of Psychology, University of Milano-Bicocca, Milan, Italy; 3 Neuropsychological Laboratory, Department of Neurorehabilitation Sciences, IRCCS Istituto Auxologico Italiano, Milan, Italy

**Keywords:** Aggression, Sex, Dorsolateral prefrontal cortex, Transcranial direct current stimulation, Interhemispheric imbalance

## Abstract

A growing body of evidence has shown the key role of the dorsolateral prefrontal cortex (dlPFC) in aggressive behavior, along with the chance of modulating it by means of transcranial direct current stimulation (tDCS). However, the functional interplay between the two cerebral hemispheres in the regulation of aggressive behavior is still unclear. To address this issue, we assessed the effect of bi-hemispheric prefrontal tDCS in 76 healthy adults with a cross-over, double-blind, sham-controlled design. Half of the participants received the anodal stimulation over the right dlPFC and the cathodal stimulation over the left dlPFC (right anodal/left cathodal; Experiment 1), whereas the other half received the anodal stimulation over the left dlPFC and the cathodal stimulation over the right dlPFC (right cathodal/left anodal; Experiment 2). During tDCS, participants underwent the Point Subtraction Aggression Paradigm. All participants were also given self-report questionnaires measuring individual levels of aggression, impulsivity, and empathy to test whether these constructs were associated with the neuromodulation of aggressive responses at the Point Subtraction Aggression Paradigm. Results show a significant increase in aggressive reactions to provocation during right anodal/left cathodal prefrontal tDCS only within males, highlighting a sex-specific effect of the prefrontal neuromodulation that is also associated with individual levels of aggression. These findings provide a new insight into the brain mechanisms that regulate aggressiveness, their sex differences, and their association with dispositional aggressive tendencies.

## Introduction

Some psychiatric and neurological disorders show an increased risk of aggressive behavior, which negatively affects their treatment and care, the safety of both caregivers and patients themselves, the duration of hospitalization, and the social and familiar reintegration process (Girasek et al., 2022). A high prevalence of aggressive behavior is also present among prison inmates and forensic psychiatric patients (Gendreau et al., 1997; Greer et al., 2020; Steinert, 2002), posing substantial management problems. From a broader perspective, aggressive and violent behavior constitutes a major social and health burden throughout the world, with significant costs to society (World Health Organization, 2014). It is, therefore, of great relevance to understand the neural mechanisms underlying the regulation of aggression to guide the development of effective strategies for its prevention and management in clinical and forensic settings.

Different theoretical models of aggression have been proposed so far, most of them emphasizing the distinction between reactive and proactive aggressive behavior (Dodge, 1991; Crick & Dodge, 1996; Schwartz et al., 1998). Reactive aggression refers to acts typically committed in response to provocation, driven mainly by impulsive behavior and negative affective states, such as frustration and/or anger (Bertsch et al., 2020). On the other hand, proactive aggression refers to planned and goal-oriented acts aimed at obtaining benefits and/or rewards in the absence of provocation (Zhu et al., 2019).

Aggressive behavior is influenced by many different factors, including genetic predisposition (Pavlov et al., 2012; Popova, 2008), environmental influences (Liu et al., 2013; Reif et al., 2007), and sociodemographic and clinical characteristics in the case of psychiatric patients (Girasek et al., 2022). Among them, sex represents a main predictive factor of differences in the incidence and manifestation of behavioral aggression, with higher levels of both physical and verbal aggression in men as compared to women (Archer, 2004; Lightdale & Prentice, 1994; Tieger, 1980). In this regard, the meta-analytic review by Archer (2004) showed that, overall, sex differences in favor of males were greater for overt manifestations of aggression, such as physical aggression, smaller for verbal aggression, absent for anger, and either in favor of females or absent in the case of indirect forms of aggression (i.e., harming a target by rejection or exclusion). The sex difference encountered in physical aggression shows early-onset, consistent with the position of evolutionary developmental psychologists (Björkqvist & Niemela, 1992; Maccoby & Jacklin, 1974), while indirect aggression peaks during late childhood and adolescence (Björkqvist, 1994).

Dispositional tendencies such as the level of empathy, callous-unemotional traits, and impulsiveness play a significant role in the emergence of aggressive behavior. Evidence suggests that empathy may act as a mitigating factor in aggression both in the healthy population and in individuals with pronounced aggressive traits (Miller & Eisenberg, 1988). The literature has also largely documented how callous-unemotional traits are a unique and robust predictor of future offending, even after controlling for several other well-established risk factors, such as prior offending, substance use, and employment status (Pardini et al., 2006). Impulsivity and self-control play a main role in aggression as well. A recent meta-analysis (Bresin, 2019) showed that each facet of impulsivity correlates with several forms of aggressive behavior. In particular, negative urgency and lack of premeditation show significantly stronger associations with aggression than sensation seeking and lack of perseverance. Furthermore, reactive aggression is reported to be strongly associated with reactive types of impulsiveness. Consistent with these findings, another recent study (Meidenbauer et al., 2024) showed that self-reported motor impulsivity in healthy participants is highly correlated with the tendency to react aggressively following a provocation, even if such a reaction implies a financial cost. Indeed, participants with higher levels of impulsivity are more likely to seek revenge after provocation even when it would have been more advantageous to ignore it, exhibiting poor impulse control.

With respect to the neural underpinnings, several brain regions have been implicated in the regulation and expression of aggressive behavior (Raine & Yang, 2006). When a threatening or provocative stimulus is encountered, the amygdala activates downstream brain regions involved in the expression of aggression, leading to an immediate and impulsive reaction (Coccaro et al., 2007; Pardini et al., 2014). In contrast, the prefrontal cortex, through its connections with the limbic system, exerts top-down control over impulsive and aggressive tendencies (Giancola, 1995). In adults, callous-unemotional and psychopathic traits have been linked to specific neural correlates, including reduced grey matter volume and decreased activity in the amygdala (Glenn et al., 2009; Pardini et al., 2014), prefrontal cortex, and temporal gyri. These traits are also associated with disrupted white matter integrity in the dorsal cingulum and uncinate fasciculus, as well as dysfunctions in the default mode network (Johanson et al., 2020).

A key area of the cerebral network of aggressive behavior is the dorsolateral prefrontal cortex (dlPFC), given its involvement in executive functions, inhibitory control, and moral reasoning (Fumagalli & Priori, 2012; Kim & Lee, 2011). The dlPFC, together with the orbitofrontal cortex (OFC) and the ventromedial prefrontal cortex (vmPFC), forms a complex neural circuit that integrates emotional and cognitive information to guide behavioral responses (Giancola, 1995; Phillips et al., 2008). Dysfunctions of the dlPFC or of its connectivity with other brain regions, including the limbic system, can disrupt the balance between emotional reactivity and cognitive control, because it is engaged in response inhibition and selection and in the ability to correctly and promptly assess the consequences of behavior (Hoppenbrouwers et al., 2013; Repple et al., 2017). Importantly, this area was shown to be involved in both proactive and reactive behavioral control (Braver et al., 2009; Chambers et al., 2009; Vanderhasselt et al., 2014) and, therefore, in the pathogenesis of aggressive behavior as well (Cristofori et al., 2016).

Neuroimaging and neuropsychological data also suggest a functional lateralization of the prefrontal cortex during aggressive responses. In particular, the motivational direction model of frontal asymmetry (Sutton & Davidson, 1997) proposes that both anger and aggression can be conceived as consequences of frontal activity asymmetry, particularly hyperactivation of the left prefrontal cortex and/or hypoactivation of the right one. Accordingly, higher frontal excitability in the right compared to the left hemisphere was found to promote avoidance-related motivational behavior, in contrast with approach-related aggressive tendencies, associated with relative left frontal cortical activity (Hortensius et al., 2012; Schutter et al., 2008). A meta-analysis by Yang and Raine (2009) has instead highlighted an association between structural and functional alteration of the left dlPFC and antisocial behavioral features, such as scarce behavioral control, impulsivity, and response inhibition, therefore suggesting that the decreased functioning of the left dlPFC may result in antisocial disorders (Yang & Raine, 2009). Along the same line, a transcranial magnetic stimulation (TMS) and electroencephalography (EEG) co-registration study by Hoppenbrowers et al. (2013) has revealed impaired cortical inhibition within the left dlPFC in male psychopathic offenders. On the other hand, the left dlPFC has been shown to be significantly activated in aggressive responses. Its morphological features can predict aggressive tendencies, in particular proactive aggression (Gong et al., 2023; Wei & Xia, 2023; Zhu et al., 2019, 2022), whereas an activation of the right dlPFC is commonly observed during response inhibition tasks (Chambers et al., 2009). However, proactive and reactive control have been associated with both right (Paxton et al., 2008) and left (MacDonald III & Carter, 2003) dlPFC activity, and results from a recent meta-analysis of neuroimaging data has questioned the prefrontal hypoactivity model of reactive aggression (Nikolic et al., 2022). In summary, although the regulatory role played by the dlPFC on aggressive and impulsive responses is clear, evidence about the alleged hemispheric lateralization of this cortical area in aggression is still conflicting.

Considering the significant prefrontal involvement in the control of aggressive behavior, some studies have investigated the potential of noninvasive brain stimulation (NIBS), particularly transcranial direct current stimulation (tDCS), for modulating aggressive responses, in turn gaining further insight into the underlying functional mechanisms of aggression (Gilam et al., 2018; Knehans et al., 2022; Sergiou et al., 2022; Volpe et al., 2022; Weidler et al., 2022). Transcranial direct current stimulation is a neuromodulatory NIBS technique that, by influencing the resting membrane potential of neurons, can be used to increase or decrease cortical excitability. In general, cortical excitability enhancement can be induced by applying anodal stimulation, while a reduction of cortical excitability can be obtained by applying cathodal stimulation, although the behavioral effects of tDCS may have a different direction given their dependence on individual state, stimulation, and network activity (Brunoni et al., 2012; Fertonani & Miniussi, 2017; Nitsche et al., 2008).

In relation to aggressive behavior, measured with many different experimental paradigms, NIBS studies have shown that—overall—stimulating the dlPFC can modulate aggressive responses, enhancing or reducing them, but again with mixed findings with respect to putative hemispheric asymmetries (Casula et al., 2023; Knehans et al., 2022; Sergiou et al., 2020). In general, a decrease in aggression is most likely to occur via the excitatory stimulation of the right prefrontal areas, whereas increased aggression is more likely to occur by increasing the excitability of the left prefrontal cortex—in accordance with the Sutton and Davidson (1997) model. A review by Casula et al. (2023) has reported that 6 of 11 brain stimulation studies on healthy individuals—ten of which employed tDCS—obtained a decrease in aggressive behavior (Chen, 2018; Choy et al., 2018; Dambacher et al., 2015a; Gilam et al., 2018; Riva et al., 2015, 2017), three studies observed an increase (Gallucci et al., 2020; Hortensius et al., 2012; Perach-Barzilay et al., 2013), and two studies did not find any effect (Dambacher et al., 2015b; Ling et al., 2020). It was also reported that the anodal stimulation of the vlPFC, dlPFC, or mPFC of the left hemisphere could increase aggression (2/2 experiments), whereas the same stimulation applied to the right hemisphere decreased aggression (6/8 experiments). In both cases, this happened regardless of the position of the cathodal electrode. More recently, Choy & colleagues (2023) reported an effect of multisession tDCS on aggressive behavior within a sample of healthy adults, but only in male participants: after three sessions of anodal tDCS over the vmPFC, males engaged in lower levels of aggression compared with females (Choy et al., 2023).

However, drawing definitive conclusions is not possible, considering the methodological heterogeneity of aggression measures (self-report questionnaires and computerized tasks), the types of NIBS used, and even the generally small sample sizes. Moreover, the majority of NIBS studies on aggression have focused on either individual differences (Molero-Chamizo et al., 2019) or the laterality of the stimulation (Perach-Barzilay et al., 2013) without further integrating these two crucial aspects.

Within this framework, the present study explored whether and how the dlPFC of both hemispheres contributes to aggressive responses, focusing on their functional interhemispheric interplay. For this purpose, we have investigated the effect of bi-hemispheric prefrontal tDCS, which allows the modulation of interhemispheric interactions and the concurrent manipulation of the excitability of both the left and right prefrontal areas (Batista et al., 2015; Boggio et al., 2008; Bolognini et al., 2011a; Dambacher et al., 2015b; Hortensius et al., 2012; Molero-Chamizo et al., 2019). In this way, we could assess how altering the interhemispheric prefrontal balance can modulate aggressive responses.

We also took into consideration differences between males and females. In fact, sex-related brain differences have been documented at various levels, such as total brain volume, neural tissue density, and cortical asymmetry between the left and right hemispheres (Good et al., 2001; Kovalev et al., 2003). These brain differences have been shown to impact NIBS effects, including the neuromodulation of aggressive behavior (Bhattacharjee et al., 2022; Rudroff et al., 2020). In a recent study by Gallucci & colleagues (2020), for instance, anodal tDCS over the ventrolateral prefrontal cortex (vlPFC) significantly reduced aggressive differences between males and females within healthy participants, with females becoming as aggressive as males following active tDCS, regardless of the laterality of the stimulation. In addition, a study by León & colleagues (2020) also demonstrated sex-specific effects of anodal tDCS over the right OFC in a decision-making task; women had a significant increase in their performance after the stimulation and men showed no differences between active and sham tDCS. These results suggest that behavioral outcomes of neuromodulation are related to sex differences and that they might be particularly relevant in those populations in which aggression and risky decision-making are prominent (Bell et al., 2022).

Against this background, the rationale of the present study was to compare the effects of two types of bi-hemispheric prefrontal neuromodulation using the Point Subtraction Aggression Paradigm (PSAP) to evaluate whether and in which direction prefrontal interhemispheric imbalance may affect aggressive behavior. We expected to find a modulation of aggressive responses with the anodal stimulation of the right dlPFC and the concurrent cathodal stimulation of the left dlPFC (i.e., eighnt 1), as well as by reversing the stimulation, with the cathodal stimulation of the right dlPFC plus anodal stimulation of the left dlPFC (i.e., Experiment 2) but with behavioral effects going in opposite directions. According to the motivational model of frontal asymmetry (Sutton & Davidson, 1997), our hypotheses were as follows: H1) reduced aggression following right anodal/left cathodal tDCS (Experiment 1); H2) increased aggressiveness with the opposite electrodes montage (right cathodal/left anodal) (Experiment 2). Moreover, we investigated the influence of sex and personal dispositions on the neuromodulation of aggressive responses. In this case, we hypothesized a greater reduction of aggressive responses induced by tDCS in participants with higher levels of aggression and impulsiveness and the opposite outcome in the case of high levels of empathy (H3). Finally, we expected a greater effect of tDCS in male participants in terms of a greater decrease in aggressive responses (H4).

## Methods

### Participants

The sample size estimation was calculated by means of an a priori power analysis (effect size *f* = 0.25; statistical power = 0.8; alpha error level *p* = 0.05) using the software G*Power 3.1 (Faul et al., 2009), which showed a recommended sample size of at least 34 participants per experiment to achieve enough statistical power.

To face possible dropouts, we recruited more subjects than the minimum number of the recommended sample size, and we stopped data collection when we ensured we had at least 34 participants per group with both sessions. In the end, 82 healthy participants were recruited for the study; six of them did not complete the second session of the experiment and were then excluded from the analysis. Thus, the final sample consisted of 76 participants, 38 for each experiment (Experiment 1: 21 females, mean age ± standard deviation = 23.6 ± 3.1 years, mean education = 15.6 ± 2.2 years; Experiment 2: 17 females, mean age = 24.2 ± 2.7 years, mean education = 16 ± 2 years).

All participants were white/Caucasian, right-handed and with normal or corrected-to-normal vision. They all provided their written informed consent prior to the experiment. The study protocol was approved by the Ethical Committee of the University of Milano-Bicocca and in concordance with the Declaration of Helsinki. Demographic characteristics of the sample and scores obtained at the self-report questionnaires are reported in Table 1.

**Table 1:** Demographic characteristics and self-report questionnaires’ scores of the sample of Experiments 1 and 2

R-Anod/L-Cath (*Exp. 1*)		Males (*N* = 17)	Females (*N* = 21)	*t*-value	*p*-value
	Age (years)	22.9 ± 2.93	24.1 ± 3.1	− 1.28	.21
	Education (years)	15.1 ± 2.14	16 ± 2.27	− 1.24	.22
	BPAQ	71.7 ± 19.56	58.4 ± 12.24	2.57	.02*
	BIS-11	57.5 ± 8.33	54.6 ± 7.43	1.14	.26
	IRI	93.5 ± 9.61	99.7 ± 8.42	− 2.10	.04*
**R-Cath/L-Anod (*Exp. 2*)**		**Males (*N* = 21)**	**Females (*N* = 17)**	***t*-value**	***p*-value**
	Age (years)	24.2 ± 2.8	24.3 ± 2.59	− .12	.91
	Education (years)	15.9 ± 2.22	16.2 ± 1.78	− .48	.63
	BPAQ	64.1 ± 13.41	63.2 ± 11.93	.22	.83
	BIS-11	59.8 ± 9.68	59.4 ± 12.85	.10	.92
	IRI	87.4 ± 13.08	90.2 ± 14.74	− .61	.55
**Exp. 1 vs. Exp. 2**		**Exp. 1 (*N* = 38)**	**Exp. 2 (*N* = 38)**	***t*-value**	***p*-value**
	Age (years)	23.6 ± 3.05	24.2 ± 2.68	-.99	.32
	Education (years)	15.6 ± 2.23	16 ± 2.01	-.92	.36
	BPAQ	64.3 ± 17.07	63.7 ± 12.61	.18	.43
	BIS-11	55.9 ± 7.87	59.6 ± 11.05	−1.67	.05*
	IRI	96.9 ± 9.37	88.7 ± 13.72	3.07	.01*

All data are reported with mean ± standard deviation. BPAQ = Buss and Perry's Aggression Questionnaire; BIS = Barratt Impulsiveness Scale; IRI = Interpersonal Reactivity Index. Data were compared by means of independent samples *t*-tests; *Significant difference

### Materials and methods

#### Transcranial Direct Current Stimulation

**Fig. 1:**
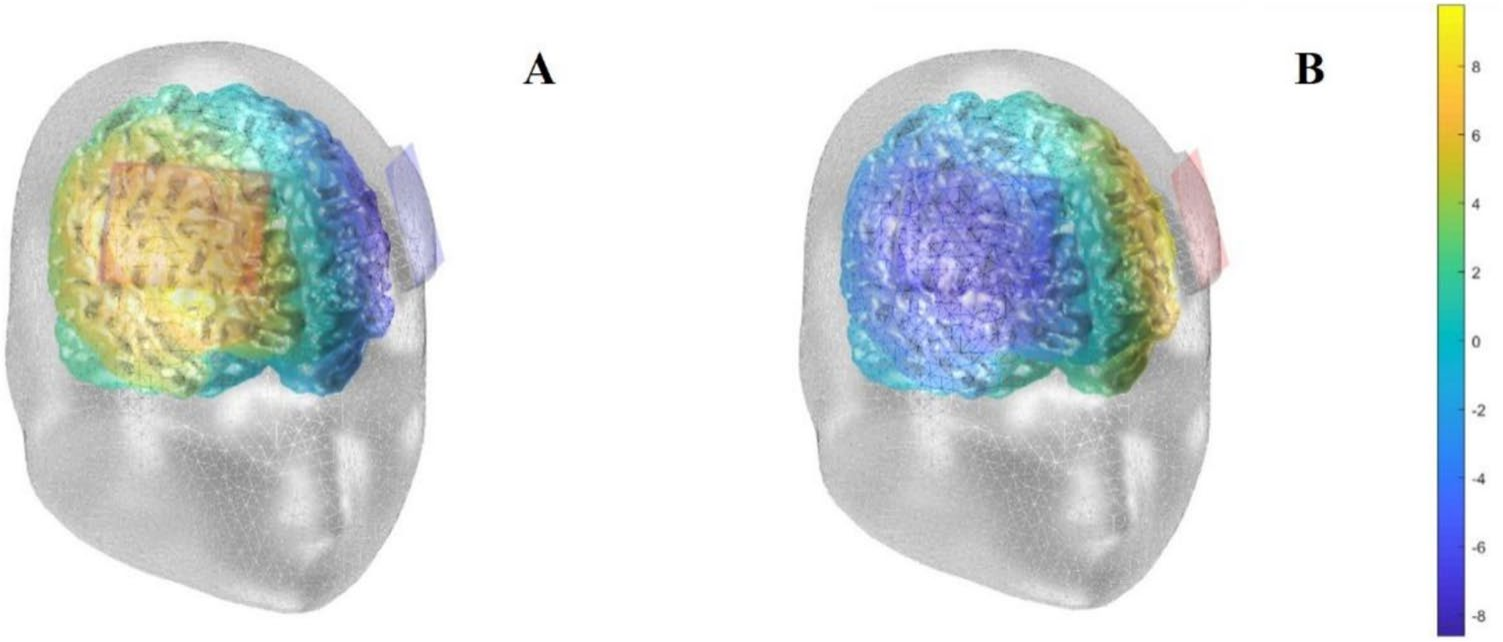
Simulation of tDCS. The simulation of the electrical currents in Experiments 1 (**A**) and 2 (**B**) performed by using the “Comets2” toolbox (Lee et al., 2017). The simulation was made by placing the anode over the right dlPFC in Exp. 1 vs. left dlPFC in Exp. 2, while the cathode was placed over the contralateral homologous area. The color bar indicates the simulated electric potential (V): yellow = positive, dark blue = negative

The tDCS was administered with the BrainSTIM stimulator (EMS s.r.l, Bologna, Italy; emsmedical.net). The BrainSTIM stimulator is programmable and allows the management of real or placebo stimulation protocols, which were activated with codes for conducting double-blind studies. The tDCS protocol involved the use of two electrodes (anode and cathode), each of 5 × 7 cm. After being placed in two saline-soaked sponges, accordingly to the experimental group (see below), one electrode (anode or cathode) was placed on the scalp over the right or left dlPFC (F3 or F4), while the other electrode was placed over the contralateral dlPFC, following the international EEG 10–20 system for electrodes placements (Fig. 1). The real stimulation consisted of the continuous application of a current at an intensity of 1.5 mA for 25 min (fade-in/fade-out phases = 30 s). The same stimulation was used for sham tDCS, but the stimulator was turned off after 30 s to ensure that participants could feel the initial sensations at the beginning of the stimulation, a requisite for successful masking (Thair et al., 2017). In both cases, tDCS started 3 min before the experimental task, which was performed during stimulation. The tDCS device was set in advance to deliver real or sham tDCS, thus keeping both the participant and the experimenter blinded to tDCS allocation.

At the end of each tDCS session, participants were administered a 7-item questionnaire (adapted from Fertonani et al., 2015) to assess the side effects of tDCS. No serious adverse events were reported by participants; only some mild and transient side effects (e.g., itching, dizziness, and tingling) were described in a limited number of participants.

#### Point Subtraction Aggression Paradigm

To measure aggression, we used the PSAP, a well-validated computerized paradigm measuring aggressive behavior (Cherek, 1981). It consists of an online game, administered through a PC, in which participants compete against a fictitious opponent, who they believe is real and sitting in the room next door. To ensure that participants believe they are playing against someone else, a loading screen is displayed on the monitor for 3 s before the beginning of the game, showing the following text: “Establishing connection to the other player.…” Subjects are informed that the goal is to earn as many points as possible by pressing a certain key on the computer keyboard a certain number of consecutive times. Specifically, to gain one point, participants have to press the “A” key on the keyboard 100 consecutive times. Subjects are also informed that they may see their point total decrease, because the other player is subtracting points from them. Participants are told that they can react to this provocation in three ways: continue to earn points by pressing the same key (A); start pressing a second key (B) 10 consecutive times, which allows them to subtract points from the opposing player; or press a third key (C) 10 consecutive times to protect their points from the opponent’s steals for a certain amount of time. Time interval is random; it can range from 1 to 6 s. Subjects are informed that they have been randomly assigned to the group in which they cannot retain any points subtracted from the other participant. Therefore, because stealing is of no benefit to the subject, and given that it represents a behavior directed toward the goal of harming someone who would prefer to avoid such treatment, it is consistent with Baron and Richardson’s (1994) definition of aggression. The number of B presses is thus used as a behavioral index of aggression (i.e., SUBTRACT responses). By contrast, the number of A responses represents the gaining behavior (i.e., GAIN responses), while the number of C presses indicates the self-preserving behavior (i.e., PROTECT responses). The PSAP was shown to have high validity throughout different studies (Cherek et al., 1996, 2000; Dougherty et al., 1999; Geniole et al., 2017; McCloskey et al., 2009), and the 25-min version used here was also shown to maintain good psychometric properties (Golomb et al., 2007). Moreover, neuroimaging studies have demonstrated that the PSAP is a useful task for probing prefrontal regulatory processes (Skibsted et al., 2017). Nonetheless, it allows to measure not only aggressive responses to provocation but also self-protective or gain-oriented responses, hence permitting the evaluation of potential dissociable effects of the prefrontal stimulation.

#### Buss and Perry’s Aggression Questionnaire

The Buss and Perry’s Aggression Questionnaire (BPAQ) (Buss & Perry, 1992; Fossati et al., 2003) is a 29-item questionnaire that is widely used as a measure of self-reported aggression. Participants are asked to rate each item on a scale from 1 (extremely not characteristic of me) to 5 (extremely characteristic of me). The total score ranges from 29 to 145; higher scores represent higher levels of aggression. Moreover, four fundamental components of aggression can be derived by subscores: physical aggression (score range, 9–45), verbal aggression (range, 5–25), anger (range, 7–35), and hostility (range, 8–40).

#### Barratt Impulsiveness Scale

The Barratt Impulsiveness Scale (BIS-11) represents one of the best-known and most widely used instruments for measuring impulsivity (Version 11; Patton et al., 1995; Albiero et al., 2006). The BIS-11 is a scale that tends to synthesize the concept of impulsivity around three basic elements: 1) motor impulsiveness (range, 11–44), defined as the tendency to take immediate action as a somehow “reflex”; 2) attentional impulsiveness (range, 8–32), an expression of the tendency to initiate actions that are always new and different due to a difficulty to concentrate or focus attention and to easy distractibility; 3) nonplanning impulsiveness (range, 11–44), understood as the tendency to make decisions that are not thoughtful with respect to the short-, medium- and long-term consequences of behaviors. The scale BIS-11 consists of 30 items; each item is rated on a 4-point scale (1 = extremely uncharacteristic of me to 5 = extremely characteristic of me), for a total score ranging from 30 to 120: the greater the score, the greater the impulsivity.

#### Interpersonal Reactivity Index

The Interpersonal Reactivity Index (IRI) (Davis, 1983; Maddaluno et al., 2022) is one of the most used self-report questionnaires for measuring empathy in adults. It consists of 28 items (total score range, 28–140), divided into four subscales: fantasy (range, 7–35), perspective taking (range, 7–35), empathic concern (range, 7–35), and personal distress (range, 7–35). Each item is rated on a 4-level scale: from 1 (does not describe me at all) to 5 (describes me perfectly); consequently, higher scores reflect higher levels of emphatic abilities.

## Experimental design

A double-blind within-subjects design was used for both Experiments 1 and 2. In each experiment, every participant underwent two tDCS sessions, separated by a minimum of 3 days to a maximum of 10 days, during which they received in a randomized order real or sham tDCS.

**Fig. 2:**
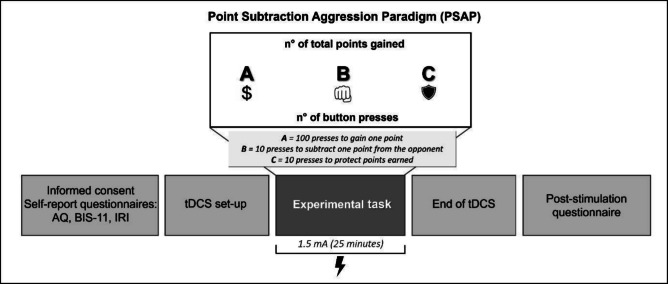
Procedure and timeline of the study for both Experiment 1 and 2

Before starting the first tDCS session, all participants completed the self-report questionnaires in the following order: BPAQ, BIS-11, and IRI. Then, instructions for the PSAP were given; only after making sure that the task was understood, the experimental session began. In both sessions, all participants underwent the PSAP while receiving tDCS. In line with previous studies (Bolognini et al., 2011b; Karuza et al., 2016; Vöckel et al., 2024), the stimulation began 3 min before the start of the task to induce a modulation of neuronal excitability within the target area before the beginning of the PSAP. In Experiment 1, the anodal stimulation was delivered over the right dlPFC, and the cathodal stimulation over the left dlPFC (right anodal/left cathodal). In Experiment 2, participants received anodal stimulation over the left dlPFC and cathodal stimulation over the right dlPFC (right cathodal/left anodal). At the end of both tDCS sessions, every participant completed the 7-item questionnaire to assess the tDCS side effects. The experimental procedure is illustrated in Fig. 2.

## Data analysis

Analyses of participants’ responses were performed by means of IBM SPSS Statistics, version 28.0 (IBM Corp, 2021) and R Studio, version 4.2.3 (R Core Team, 2021).

Descriptive statistics are reported in Table 1, along with comparisons by means of independent samples *t*-tests to verify differences between males and females with respect to age, education, and scores at the self-report questionnaires.

After checking for normality of residuals, in order to directly compare the effect of the two types of neuromodulation for each response type at the PSAP (SUBTRACT, GAIN, PROTECT), a 2 × 2 × 2 mixed repeated-measures analysis of variance (rmANOVA) was conducted, with Experiment (Exp. 1, right anodal/left cathodal vs. Exp. 2, right cathodal/left anodal) and sex (male vs. female) as the between-subjects factors, tDCS as the within-subjects factor (sham vs. active), and the percentage of key presses as the dependent variable. We also included as covariates in the model the *z*-transformed total scores of the three questionnaires we administered (BPAQ, BIS-11, and IRI) to account for personality differences between participants from Experiment 1 vs. Experiment 2, as well as the number of provocations received by the fictitious opponent. Significant Experiment x Sex x tDCS interactions were then further analysed with two separate Sex x tDCS rmANOVAs, one for each experiment, followed by post-hoc *t*-tests for paired samples.

Finally, partial correlations between the percentage of the three types of PSAP response (SUBTRACT, GAIN, PROTECT) during active tDCS and scores at the self-report questionnaires (BPAQ, BIS-11, IRI) were conducted separately for each experiment, controlling for the number of provocations and using the Benjamini–Hochberg correction to adjust for multiple comparisons.

## Results

### SUBTRACT responses at the PSAP

 The 3-levels rmANOVA showed only a significant tDCS X Experiment X Sex interaction (*F*_1,68_ = 6.27, *p* = 0.015, η_p_^2^ = 0.08); other main effects and interactions did not attain the significance level as shown in Table 2. The tDCS X Experiment x Sex interaction was then explored with two separate rmANOVAs, one for each experiment.

**Table 2:** Main effects and interactions for the 3-level rmANOVA

		*F*-value	*p*-value	η_p_^2^
SUBTRACT responses	tDCS	1.35	.25	.02
	Experiment	2.67	.11	.04
	Sex	.31	.58	0
	*Z*-BPAQ	1.48	.23	.02
	*Z*-BIS-11	.04	.85	0
	*Z*-IRI	1.01	.32	.02
	tDCS x *Z*-BPAQ	1.23	.27	.02
	tDCS x *Z*-BIS-11	3.15	.08	.04
	tDCS x *Z*-IRI	.01	.93	0
	tDCS x Experiment	.03	.87	0
	tDCS x Sex	.66	.42	.01
	Experiment x Sex	2.36	.13	.03
	**tDCS X Experiment x Sex**	**6.27**	.02*	**.08**
GAIN responses	tDCS	2.52	.12	.04
	Experiment	.4	.53	.01
	Sex	2.21	.14	.03
	*Z*-BPAQ	1.86	.18	.03
	*Z*-BIS-11	.12	.73	0
	*Z*-IRI	0	.99	0
	tDCS x *Z*-BPAQ	2.17	.15	.03
	tDCS x *Z*-BIS-11	3.06	.09	.04
	tDCS x *Z*-IRI	.01	.93	0
	tDCS x Experiment	.4	.53	.01
	tDCS x Sex	.38	.54	.01
	Experiment x Sex	.63	.43	.01
	**tDCS X Experiment x Sex**	**5.76**	.02*	**.08**
PROTECT responses	tDCS	1.31	.26	.02
	**Experiment**	**7.81**	.01*	**.1**
	Sex	1.65	.2	.02
	*Z*-BPAQ	.09	.77	0
	*Z*-BIS-11	.05	.82	0
	*Z*-IRI	1.52	.22	.02
	tDCS x *Z*-BPAQ	1.13	.29	.02
	tDCS x *Z*-BIS-11	.67	.42	.01
	tDCS x *Z*-IRI	.07	.79	0
	tDCS x Experiment	.63	.43	.01
	tDCS x Sex	.01	.93	0
	Experiment x Sex	.67	.42	.01
	tDCS X Experiment x Sex	1.05	.31	.02

*tDCS* transcranial direct current stimulation, *BPAQ* Buss and Perry's Aggression Questionnaire, *BIS* Barratt Impulsiveness Scale, *IRI *Interpersonal Reactivity Index. * *significant difference*

**Fig. 3:**
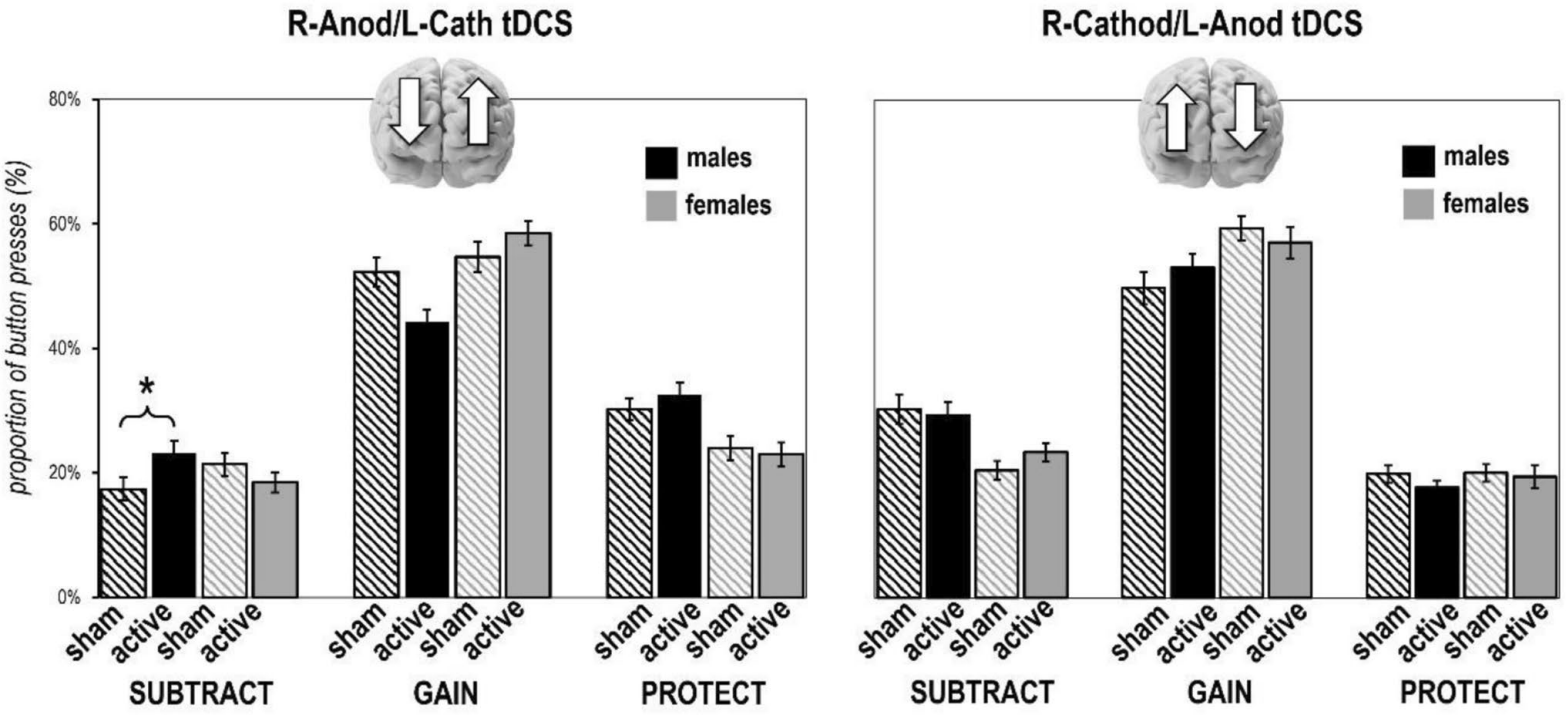
PSAP performance during active and sham R-Anod/L-Cath (Exp. 1) and R-Cath/L-Anod (Exp. 2) prefrontal tDCS. The percentage of response types at the PSAP (SUBTRACT, GAIN, PROTECT) during active and sham tDCS, with the anode over the right dlPFC and the cathode over the left dlPFC (R-Anod/L-Cath; Experiment 1) and with the cathode over the right dlPFC and the anode over the left dlPFC (R-Cath/L-Anod; Experiment 2). Striped white/black bars = males with sham tDCS; black bars = males with active tDCS; striped white/grey bars = females with sham tDCS; grey bars = females with active tDCS; error bars = standard error (SEM); *p < .05

For Experiment 1 (right anodal/left cathodal), the 2 × 2 rmANOVA showed a significant interaction tDCS x Sex (*F*_1,35_ = 5.34, *p* = 0.027, η_p_^2^ = 0.13), while the main effects of tDCS (*F*_1,35_ = 0.19, *p* = 0.665, η_p_^2^ = 0.01) and Sex (*F*_1,35_ = 0.02, *p* = 0.904, η_p_^2^ = 0.001) did not attain the significance level. Post-hoc paired *t*-tests showed a significant difference between active and sham tDCS in males (*t*_16_ = − 2.14, *p* = 0.048) but not in females (*t*_20_ = 1.07, *p* = 0.3): males acted more aggressively while receiving active tDCS (23% of SUBTRACT responses) than during sham tDCS (17%) (Fig. 3).

For Experiment 2 (right cathodal/left anodal), the rmANOVA showed no significant effects: tDCS (*F*_1,35_ = 1.26, *p* = 0.27, η_p_^2^ = 0.04), Sex (*F*_1,35_ = 3.49, *p* = 0.07, η_p_^2^ = 0.09), tDCS x Sex (B: *F*_1,35_ = 1.85, *p* = 0.182, η_p_^2^ = 0.05).

### GAIN responses at the PSAP 

The 3-level rmANOVA showed only a significant tDCS x Experiment x Sex interaction (*F*_1,68_ = 5.76, *p* = 0.019, η_p_^2^ = 0.08), as shown in Table 2. For Experiment 1 (right anodal/left cathodal), the rmANOVA showed a significant tDCS by Sex interaction (*F*_1,35_ = 6.81, *p* = 0.013, η_p_^2^ = 0.16), while the main effects of tDCS (*F*_1,35_ = 0.43, *p* = 0.514, η_p_^2^ = 0.01) and Sex (*F*_1,35_ = 1.26, *p* = 0.27, η_p_^2^ = 0.04) were not significant. Post-hoc paired *t*-tests showed a nearly significant difference between active and sham tDCS in male participants (*t*_16_ = 2.03, *p* = 0.059), but not in females (*t*_20_ = − 1.34, *p* = 0.195).

For Experiment 2 (right cathodal/left anodal), the rmANOVA did not show any significant effect: tDCS x Sex (*F*_1,35_ = 1.67, *p* = 0.205, η_p_^2^ = 0.05), tDCS (*F*_1,35_ = 2.22, *p* = 0.146, η_p_^2^ = 0.06), and Sex (*F*_1,35_ = 3.48, *p* = 0.071, η_p_^2^ = 0.09)**.**

### PROTECT responses at the PSAP

The mixed rmANOVA showed only a significant effect of Experiment (*F*_1,68_ = 7.81, *p* = 0.007, η_p_^2^ = 0.1), revealing that overall participants from Experiment 1 (right anodal/left cathodal) tended to choose PROTECT responses more frequently than participants from Experiment 2 (right cathodal/left anodal) (27% vs. 19%). Detailed results are reported in Table 2.

Although differences at the BIS-11 and IRI between the two experimental groups can be appreciated (Table 1), adding the *z-*transformed total scores as covariates in the model does not change the outcome of the stimulation on both aggressive and gain-oriented responses; hence. tDCS effects cannot be explained by differences at the questionnaires’ scores from the two samples (Experiment 1 vs. Experiment 2; Table 2).

## Correlations

### SUBTRACT responses at the PSAP

 In Experiment 1 (right anodal/left cathodal), the partial correlation analysis revealed a positive association between SUBTRACT responses during active right anodal/left cathodal prefrontal tDCS and the BPAQ hostility subscore (*r* = 0.41, *p* = 0.05). No other significant correlation emerged (all *ps* ≥ 0.07). In Experiment 2 (right cathodal/left anodal), no significant correlation was found (all *ps* ≥ 0.25).

### GAIN responses at the PSAP

 In Experiment 1 (right anodal/left cathodal), the percentage of GAIN responses during active tDCS was positively associated with the empathic concern subscore at the IRI (*r* = 0.42, *p* = 0.04). In Experiment 2 (right cathodal/left anodal), a negative correlation emerged between the BPAQ verbal aggression sub-score and the percentage of GAIN responses during active tDCS (*r* = − 0.4, *p* = 0.05). No other significant correlation was reported (all *ps* > 0.06).

### PROTECT responses at the PSAP

 In both Experiment 1 (right anodal/left cathodal) and Experiment 2 (right cathodal/left anodal), we found no significant correlation between PROTECT responses and questionnaire scores (all *ps* s both.

## Discussion

The present study investigated how neuromodulation, namely tDCS, can alter the interhemispheric prefrontal balance involved in the regulation of aggressive behavior. In more detail, we explored the contribution of the interhemispheric interplay between right and left dlPFC activity in aggressive responses to a provocation, also considering possible differences between males and females and the association with individual aggressive, impulsive, and empathic traits.

The results show that when the anodal stimulation is applied over the right dlPFC with the concurrent cathodal stimulation of the left dlPFC, males only show a significant increase in aggressive responses (i.e., SUBTRACT responses) during active tDCS compared with sham tDCS. Reversing the stimulation, namely applying the cathode over the right dlPFC and the anode over the left dlPFC, does not influence aggressive reactions at the PSAP, although a trend in the opposite direction can be appreciated. Self-protective and gain-oriented responses, instead, are unaffected by the bi-hemispheric prefrontal stimulation of either type, except for a nearly significant trend toward a reduction of gaining responses during the excitatory stimulation of the right dlPFC and the concurrent inhibition of the left dlPFC, a tendency again only present in male participants.

These findings confirm the involvement of the prefrontal cortex in controlling aggressive reactions to provocation, showing their dependence on an interhemispheric imbalance favoring the activity of the right dlPFC along with the inhibition of the left dlPFC, as predicted by hypoactivity prefrontal models of reactive aggression (Yang & Raine, 2009). Consistent with our findings is the study by Perach-Barzilay et al. (2013), showing that the inhibitory continuous theta-burst magnetic stimulation (cTBS) of the left dlPFC increases both reactive and proactive aggressive responses in a computerized task compared with the right inhibitory dlPFC stimulation.

A possible explanation of our findings is that the anodal right prefrontal stimulation, by increasing right-to-left transcallosal inhibition, further inhibits the left dlPFC targeted with the cathodal stimulation, in turn favoring dysfunctional inhibitory neurotransmission in the dlPFC, shown to represent a key mechanism in relation to aggression (Hoppenbrouwers et al., 2013). In fact, aggression studies using mammalian models, as well as observations in human patients, have indicated that the inhibitory GABAergic system plays an essential role in modulating aggression (Nelson & Trainor, 2007).

We also need to consider how the PSAP works. This task is a reliable measure of aggression in response to provocation in a competitive environment: indeed, the reward earned depends on the performance (number of button presses) and strategy (which buttons are pressed) of the player, as well as the performance of the opponent (number of provocations). Because acting aggressively in the PSAP is costly to personal earnings, the performance may be influenced by poor (cost–benefit) decision-making (Geniole et al., 2017). In this regard, the present findings are in line with evidence showing that the bi-hemispheric neuromodulation of dlPFC excitability can influence decision-making for risky losses and gains (Pripfl et al., 2013; Ye et al., 2015).

The observed sex differences in prefrontal tDCS outcomes are likely mediated by structural and functional brain factors (Garavan et al., 2006; Luders et al., 2004; Zhang et al., 2020), which directly affect tDCS-induced neurophysiological effects in males and females (Bhattacharjee et al., 2022; Kuo et al., 2006; Rudroff et al., 2020; Vergallito et al., 2022). Additionally, PSAP aggression scores were shown to be linked to fluctuations in testosterone (Geniole et al., 2017). Worth noting, in the present study, males report a higher level of aggression (i.e., BPAQ) than females, in line with many previous studies (Buss & Perry, 1992; Fossati et al., 2003; Gerevich et al., 2007; Harris, 1995). Conversely, in our sample, females are shown to be more empathic (i.e., IRI) than males, again in line with previous evidence (Gilet et al., 2013; Maddaluno et al., 2022; Yang & Kang, 2020). Besides these differences, of greater interest is the positive correlation between the predisposition toward aggressive behavior—hostility in particular—and the increase of aggressive responses at the PSAP during prefrontal (right anodal/left cathodal) tDCS, which suggests a close relationship between individual inclination to aggression and the chance of modulating aggressive reactions. Contrarily to our initial hypotheses, the higher the individual level of hostile behavior, the more subtractions the participants inflicted on the opponent during the active—but not sham—prefrontal tDCS. Additionally, we found a positive association between the level of empathic concern and responses aimed at gaining during active tDCS. Taken together, this evidence indicates that the efficacy of bi-hemispheric prefrontal tDCS in modulating reactive aggression in males and females is related not only to their brain differences but also to their differing dispositions of aggression and empathy, the former greater in men, the latter in women. In fact, it has been reported that empathy might have a “protective role” on aggressive responses (Miller & Eisenberg, 1988), whereas the presence of callous-unemotional traits may constitute a predictor of higher aggressive and antisocial behavior, particularly in males (Pardini , 2006).

With respect to the absence of significant behavioral effects when the left dlPFC was targeted with anodal stimulation, while the homologue area in the right hemisphere was targeted with cathodal stimulation, we can only observe that, in this sample, no differences between males and females were found with respect to aggression and empathy. There is only a negative correlation between the BPAQ verbal aggression subscore and the percentage of gaining responses during active tDCS, suggesting that this component of aggressive behavior could be involved in its modulation as well. Another noteworthy result is that participants who received left anodal plus right cathodal prefrontal tDCS were less prone to choose self-protecting responses (Experiment 2) than participants who received the reversed bi-hemispheric stimulation (Experiment 1). Therefore, the absence of significant tDCS effects during left anodal and concurrent right cathodal prefrontal tDCS could be due, again, to a difference in the participants’ response style.

Overall, our study provides further evidence that personal dispositions may counteract the behavioral effects of neuromodulation: as also highlighted by Bell et al. (2022), individual differences and personality traits play an essential role in response variability to tDCS. In fact, Bell et al. (2022) showed that anodal tDCS over the right vlPFC leads to an increase in impulsive responses at a Stop Signal Task specifically in participants characterized by strong sensation seeking and lack of premeditation.

There are some limitations to the study that should be considered. First, the PSAP does not clearly differentiate between reactive and proactive aggression. Because participants can subtract points from the fictitious opponent whenever they want, and not only after a provocation has occurred, the distinction between retaliatory and instrumental acts of aggression might be blurred. Second, credibility in the task (i.e., whether participants believed or not the opponent was someone sitting in the room next door) was not assessed. Even though it has been previously reported that suspicion regarding the existence of the opposing player did not seem to influence the results at the PSAP (Geniole et al., 2017), this aspect should be thoroughly assessed in the future, considering its potential influence on behavioral responses. Lastly, the study has been conducted on a sample of healthy participants, who—even though high heterogeneity can be observed in terms of aggressive tendencies at both a behavioral and a self-reported level—disclosed no pathological level of aggression, nor this was detected by the questionnaires administered. Therefore, the chance of modulating aggressive responses by means of tDCS might be limited under healthy conditions; it would be interesting to know whether there are differences in the possibility of modulating clinical aggression, either at a symptomatologic level or even using experimental behavioural measures, such as the PSAP task. Conversely, within the clinical population more intensive stimulation over several days is likely to be necessary. In any case, the results of this study are of interest for implementing new therapeutic interventions employing tDCS.

## Conclusions

Bi-hemispheric prefrontal tDCS, used to increase the excitability of the right dlPFC and simultaneously decrease the excitability of the homologue in the left hemisphere, facilitates aggressive reactions to provocation in males, but not in females, in relation to their personality traits, particularly their aggressiveness profile. These results reinforce the importance of developing individualized neuromodulation approaches by implementing personalized treatments via neuroscience-informed stimulation parameters (Bikson et al., 2012; Fang et al., 2023; Medaglia et al., 2020). Particularly, we want to highlight to what extent, including the potential influence of sex and personal disposition in the clinical perspective, may increase a better understanding of how aggression is regulated to be able to act accordingly. By deepening our understanding of individual traits and factors, which are bound to increase heterogeneity at the interindividual level (Vergallito et al., 2022), we may focus on optimizing tDCS protocols to enhance their effectiveness. This approach could be crucial to make more targeted and functional decisions, especially in developing therapeutic interventions for clinical conditions characterized by aggressive or violent behavior (e.g., personality disorders, schizophrenia, dementia), as well as for its forensic application.

## Data Availability

Data and materials used in the study are available at https://osf.io/asvgj/.

## Code availability

Script for the Italian version of the Point Subtraction Aggression Paradigm (PSAP) will be made available to researchers upon reasonable request.

## Open practices statement

Data and materials used in the study are available at https://osf.io/asvgj/ and the experiment was open-ended registered (registration DOI: https://doi.org/10.17605/OSF.IO/4MFC8).
